# Molecular Mechanisms of Cardiac Fibrosis: A Pathologist’s Perspective

**DOI:** 10.3390/cimb48030278

**Published:** 2026-03-05

**Authors:** Andrea Marzullo, Cecilia Salzillo

**Affiliations:** 1 Department of Precision and Regenerative Medicine and Ionian Area, Pathology Unit, University of Bari “Aldo Moro”, 70124 Bari, Italy; cecilia.salzillo@uniba.it; 2 Department of Experimental Medicine, PhD Course in Public Health, University of Campania “Luigi Vanvitelli”, 80138 Naples, Italy

**Keywords:** cardiac fibrosis, myofibroblast activation, TGF-β/SMAD signaling, extracellular matrix remodeling, molecular pathology

## Abstract

Cardiac fibrosis represents a final common pathway in a wide range of cardiac disorders, leading to structural remodeling, diastolic dysfunction, and heart failure. From a pathologist’s viewpoint, fibrotic remodeling displays distinctive morphologic patterns such as interstitial, perivascular, and replacement fibrosis, which mirror specific cellular and molecular mechanisms. Central to this process is the activation of cardiac fibroblasts into myofibroblasts, driven by profibrotic signaling cascades such as transforming growth factor beta (TGF-β)/mothers against decapentaplegic homolog proteins (SMAD), Wingless/Integrated signaling pathway (Wnt)/βeta-catenin, and Hippo-Yes-associated protein (YAP)/transcriptional coactivator with PDZ-binding motif (TAZ) pathways. Neurohumoral mediators, including angiotensin II and aldosterone, further amplify extracellular matrix synthesis and tissue stiffness. Epigenetic modulators and non-coding RNAs (n-c RNAs) orchestrate transcriptional programs that perpetuate fibroblast activation. Histopathological correlates of these molecular events, collagen deposition, alpha-smooth muscle actin (α-SMA) expression, and extracellular matrix (ECM) cross-linking, can be demonstrated through immunohistochemistry and digital morphometry. This review integrates molecular signaling and morphologic evidence to delineate the mechanisms of cardiac fibrosis, emphasizing the pathologist’s role as a link between molecular insight and diagnostic interpretation. Understanding these intertwined processes provides the foundation for novel antifibrotic therapies targeting key molecular nodes of fibroblast activation and matrix remodeling.

## 1. Introduction

Cardiac fibrosis is a pathological process characterised by excessive deposition of ECM proteins within the myocardium, particularly collagen, which alters the normal tissue architecture and three-dimensional organisation of cardiac tissue. This phenomenon involves expansion of the cardiac interstitium and may manifest as interstitial, perivascular, or replacement fibrosis in areas of tissue injury, with a significant impact on cardiac function [1].

Clinically, fibrosis represents a central component of pathological cardiac remodelling, encompassing a series of structural changes that occur in response to mechanical stress, chronic inflammation, or ischaemic injury and that often progress to diastolic dysfunction and heart failure. Stiffening induced by excessive ECM accumulation increases ventricular filling pressures and impairs normal diastolic filling kinetics, thereby contributing to the development of heart failure with preserved ejection fraction (HFpEF) in a substantial proportion of patients with heart failure [2,3].

The importance of integrating morphological data with molecular biology approaches is now widely recognised. Conventional histopathological analysis, which identifies patterns of collagen deposition, fibroblast activation, and structural tissue alterations, must be complemented by an understanding of the underlying cellular and molecular mechanisms, including activation of profibrotic signalling pathways and the phenotypic plasticity of cardiac fibroblasts. This integrated approach facilitates the identification of biomarkers, potential therapeutic targets, and correlations between histological features and molecular phenotypes revealed by state-of-the-art omics technologies [4,5,6].

The aim of this review is to correlate the classical histopathological patterns of cardiac fibrosis with the principal cellular and molecular mechanisms responsible for their development, highlighting how these interactions influence the pathophysiology of cardiac remodelling and its associated clinical manifestations. This integrated perspective is intended to support diagnostic practice and to inform the future development of more targeted therapeutic strategies.

## 2. Materials and Methods

This manuscript is a narrative review of the literature.

Study selection was conducted through a non-systematic search of major biomedical databases, such as PubMed/MEDLINE, Scopus, and Web of Science, including articles published between 2020 and 2025.

The keywords used included combinations of terms such as “cardiac fibrosis,” “myocardial fibrosis,” “cardiac fibroblasts,” “myofibroblasts,” “extracellular matrix,” “TGF-β,” “Wnt/β-catenin,” “YAP/TAZ,” and “epigenetic regulation.”

Original articles and reviews addressing the molecular, cellular, and histopathological mechanisms of cardiac fibrosis were included, with particular attention to studies integrating experimental data with diagnostic and clinical implications. Case reports, preliminary communications, editorials, and studies not specifically focused on myocardial fibrosis were excluded.

The histological images are derived from archived human myocardial tissue samples collected at autopsy and routinely processed in the Pathology Unit. Tissue samples were fixed in 10% neutral-buffered formalin, paraffin-embedded, sectioned at 3–4 µm thickness, and stained with Hematoxylin-Eosin and Masson’s trichrome according to standard diagnostic protocols.

## 3. Morphological Aspects of Cardiac Fibrosis

### 3.1. Main Types of Fibrosis

Cardiac fibrosis presents a variety of histopathological patterns based on the distribution of the extracellular matrix and the etiopathogenetic context. A commonly adopted classification distinguishes interstitial fibrosis, perivascular fibrosis and replacement fibrosis, each with specific morphological characteristics and functional significance [7,8].

Interstitial fibrosis is characterized by diffuse accumulation of collagen in the interstitial space between cardiomyocytes, without massive myocardial cell loss. This ECM deposition expands the endomyosial and perimyosial interstitium, altering the normal tissue architecture and creating a “fibrotic space” that can be observed histologically with collagen-specific stains. Structural alterations include an increase in collagen fiber thickness and a distortion of myofibrillar alignment, aspects that can also be quantified by digital morphometry [9,10].

**Figure 1:**
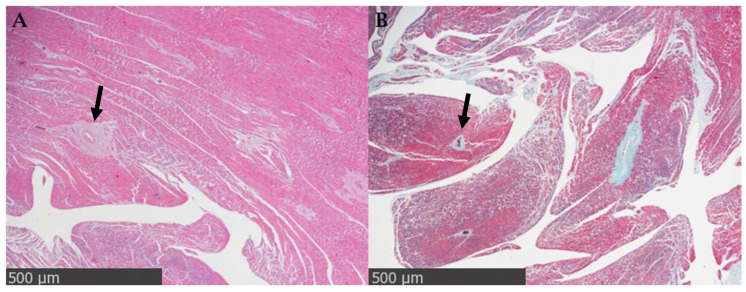
Perivascular fibrosis in adult human myocardium (47-year-old individual). Representative images of original observations obtained from formalin-fixed, paraffin-embedded left ventricular myocardial tissue collected at autopsy. (**A**) Hematoxylin-Eosin staining shows concentric accumulation of extracellular matrix around an intramyocardial vessel (black arrow; scale bar: 500 µm; original magnification 20×). (**B**) Masson’s trichrome staining shows perivascular collagen deposition (black arrow; scale bar: 500 µm; original magnification 20×).

Perivascular fibrosis (Figure 1) is defined by the accumulation of collagen and other ECM components around intramyocardial coronary vessels, particularly in the tunica adventitia of small intramyocardial vessels. This pattern is frequently observed in conditions of chronic hypertension or increased hemodynamic stress, where neurohormonal stimulation and mechanical factors contribute to the activation of perivascular fibroblasts. The accumulation of ECM around the vessels can limit the flow of oxygen and nutrients to the surrounding myocardium and promote both diastolic and microvascular dysfunction [11,12].

**Figure 2:**
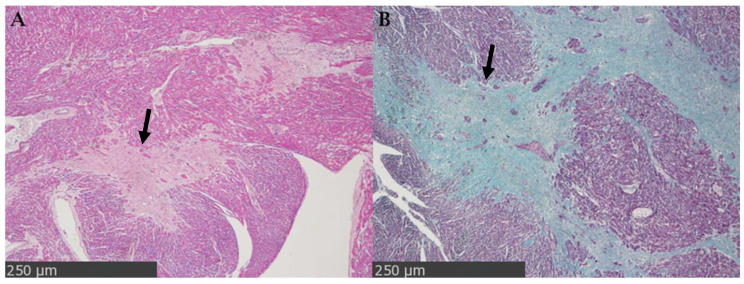
Replacement fibrosis in adult human myocardium (63-year-old individual). Representative images of original observations obtained from formalin-fixed, paraffin-embedded left ventricular myocardial tissue collected at autopsy. (**A**) Hematoxylin-Eosin staining shows replacement of necrotic myocardial tissue with a dense fibrous scar, characterized by loss of cardiomyocytes and deposition of extracellular matrix (black arrow; scale bar: 250 µm; original magnification 40×). (**B**) Masson’s trichrome staining shows extensive collagen deposition with replacement of myocardial fibers (black arrow; scale bar: 250 µm; original magnification 40×).

Replacement of reparative fibrosis (Figure 2) represents a repair process that follows necrosis or apoptosis of cardiomyocytes, as typically occurs after myocardial infarction or extensive tissue damage. In this case, the lost myocardial tissue is progressively replaced by a dense collagen scar, which partially restores the mechanical integrity of the heart but not the contractile function of the myocardial cells. This type of fibrosis is mainly associated with the formation of stable scars at the sites of injury and is considered a marker of irreversible myocardial damage [13,14].

The main histopathological patterns of cardiac fibrosis and their associated molecular mechanisms are summarized in Table 1.

### 3.2. Histopathological Correlates

One of the hallmarks of cardiac fibrosis is the excessive accumulation of ECM proteins, particularly type I and type III fibrillar collagen, which contributes to interstitial expansion and increased myocardial stiffness. In several cardiac pathological conditions, including chronic hypertension and chronic kidney disease, an increased collagen I:III ratio has been observed, with a prevalence of type I collagen related to increased tissue stiffening [15].

Another key histological marker in cardiac fibrosis is the presence of activated myofibroblasts, deriving from resident fibroblasts that characteristically express α-smooth muscle actin (α-SMA). These cells are responsible for the massive synthesis of ECM and its deposition in areas of remodelling, and their identification by immunohistochemistry for α-SMA is considered one of the main indicators of the fibrotic response in the myocardium [16].

Increased ECM cross-linking represents a further phenomenon associated with fibrosis. Collagen fibers are stabilized through covalent bonds mediated by enzymes such as lysyl oxidase (LOX) and its isoforms, increasing stiffness and resistance to proteolytic degradation. This process contributes to the formation of a denser and less deformable fibrotic tissue, a characteristic observed in hearts affected by advanced fibrosis [17].

Special histological stains remain essential to visualize and quantify collagen deposition. Masson’s trichrome stain highlights collagen in contrast to cellular elements, allowing a clear distinction between normal and fibrotic tissue, while Picrosirius Red staining allows excellent visualization of collagen fibrils and, when observed under a polarized light microscope, also allows a qualitative evaluation of the different types of fibers [18,19].

Immunohistochemistry represents a complementary approach to identify specific ECM components and cellular antigens such as collagen I/III, fibronectin and α-SMA, providing information on the distribution and type of fibrotic deposits [20].

Finally, digital quantitative analysis of histological sections has become an essential tool in the modern assessment of fibrosis. Using image processing software, it is possible to objectively quantify the extent of fibrosis, providing more precise measurements of collagen deposition area than visual analysis alone and reducing inter-observer variability [21,22].

Table 2 summarizes the main histological and immunohistochemical markers of cardiac fibrosis and their diagnostic relevance.

## 4. Cellular Biology of Cardiac Fibrosis

### 4.1. Origin and Role of Cardiac Fibroblasts

In the adult myocardium, resident cardiac fibroblasts represent a major non-myocyte population and play a crucial role in maintaining the ECM, synthesizing structural components such as collagen, fibronectin, and proteoglycans necessary to preserve the structure and mechanical integrity of the heart under physiological conditions [23]. These cells derive from resident populations characterized by markers such as PDGFR-α and Tcf21 and constitute the basic cellular reserve from which the fibrotic response arises in the event of pathological insult [16].

In response to injurious stimuli such as myocardial infarction, mechanical stress or inflammatory processes, fibroblasts can undergo a process of activation and trans-differentiation into myofibroblasts, cells with increased synthetic and contractile properties [24]. This cellular plasticity is one of the central events in the pathogenesis of cardiac fibrosis; in fact, myofibroblasts express typical markers such as α-smooth muscle actin (α-SMA), assume a contractile phenotype and overproduce ECM proteins, especially fibrillar collagen, contributing to the extensive matrix deposition that characterizes fibrotic tissue [25].

The myofibroblastic phenotype is characterized by a significant increase in the expression of α-SMA, which is incorporated into cytoskeletal contractile fibers and is widely used as a marker to identify these activated cells in histological sections. Myofibroblasts are responsible for the massive deposition of ECM and participate in the regulation of remodelling during the reparative and pathological phases, although their persistence in the late phase can lead to chronic accumulation of collagen and tissue stiffness associated with cardiac dysfunction [23,26,27].

Recent studies show that the cardiac fibroblast population is heterogeneous and includes subtypes with different gene expression and functional capacity, but trans-differentiation into myofibroblasts appears to be the main pathway by which these resident fibroblasts contribute to ECM synthesis and deposition in the fibrotic process [28].

### 4.2. Myofibroblast Activation

The activation of cardiac fibroblasts and their transdifferentiation into myofibroblasts represent a central step in the pathogenesis of cardiac fibrosis, and are regulated by a series of mechanical, inflammatory and neurohormonal stimuli as well as by paracrine signals from different cell populations of the cardiac microenvironment.

Mechanical and biomechanical stimuli play a key role in fibroblast activation. Changes in ECM stiffness and mechanical stress induced by increased ventricular pressure or alterations in functional load favour the transition of fibroblasts towards a highly synthetic and contractile myofibroblastic phenotype. These stresses are transduced into intracellular signals through mechano-transduction mechanisms involving pathways such as YAP/TAZ, Rho/ROCK and MAPK, promoting α-SMA expression and ECM production [29,30].

Inflammatory and neurohormonal stimuli are also crucial in promoting myofibroblast activation. After myocardial injury or under chronic stress conditions, inflammatory mediators such as interleukins (IL-1β, IL-6), TNF-α and chemokines released by damaged cardiomyocytes and activated immune cells activate profibrotic signalling pathways such as the TGF-β/Smad pathway, which is a major driver of fibroblast-to-myofibroblast transition and subsequent collagen synthesis [27,31].

Paracrine factors derived from cardiomyocytes, immune cells, and endothelial cells further contribute to the regulation of fibroblast activation. Stressed or necrotic cardiomyocytes release damage-associated molecular patterns (DAMPs) and pro-inflammatory cytokines that attract and activate macrophages and other immune cells, which in turn secrete further profibrotic stimuli promoting the differentiation of fibroblasts into myofibroblasts. Similarly, endothelial cells can release growth factors such as PDGF and TGF-β, which intensify the fibrotic response and promote the expansion of the myofibroblast population in injured cardiac tissue [31,32].

Myofibroblast activation is the result of a complex interaction between mechanical stimuli, inflammatory and neurohormonal signals, and paracrine factors produced by different cell types present in the stressed myocardium. These signals converge on intracellular transduction pathways that drive the phenotypic transition and the massive ECM production typical of cardiac fibrosis [27,31].

## 5. Molecular Signalling Pathways in Cardiac Fibrosis

### 5.1. TGF-β/SMAD Pathway

Transforming growth factor β (TGF-β), particularly the TGF-β1 isoform, is considered a master regulator of fibrogenesis in various organs, including the myocardium. Its activation promotes the transdifferentiation of fibroblasts into myofibroblasts and the induction of a profibrotic transcriptional program [33].

Engagement of the TGF-β receptor pathway involves the activation of type I/II receptors, the phosphorylation and nuclear translocation of the mediators SMAD2/3 (with modulation by SMAD4 and SMAD7), which regulate the expression of genes encoding collagen, fibronectin, and matrix-associated factors such as CTGF [34,35].

Experimental studies demonstrate that selective inhibition of TGF-β/SMAD signaling attenuates fibroblast activation and ECM deposition in myocardial injury models, confirming the causal role of this pathway in fibrosis formation [36,37,38].

### 5.2. Wnt/β-Catenin

The Wnt/β-catenin pathway contributes to the regulation of cell proliferation and differentiation in the heart. Canonical activation of the pathway stabilizes cytoplasmic β-catenin, which then translocates to the nucleus and modulates the transcription of profibrotic targets [39,40].

In cardiac fibroblasts, Wnt/β-catenin activation promotes proliferation and can support the acquisition of a synthetic phenotype, interacting with other pathways (Notch, TGF-β) and amplifying the fibrotic response. Consequently, the pathway is the subject of preclinical studies aimed at modulating fibrosis by specific inhibitors [41,42,43].

### 5.3. Hippo-YAP/TAZ Pathway

The Hippo pathway and its nuclear effectors YAP/TAZ represent a key sensor of mechanical signals and matrix stiffness; in fact, conditions of increased ECM stiffness or altered mechanical loading promote the activation of YAP/TAZ, which in turn induces pro-proliferative and pro-fibrotic transcriptional programs in cardiac fibroblasts [44,45].

Genetic and pharmacological studies indicate that modulation of YAP/TAZ in stromal cells can limit fibroblast expansion and attenuate tissue remodelling, directly linking mechanical signals to fibrosis progression [44,45].

### 5.4. Neurohormonal Mediators: Angiotensin II and Aldosterone

The renin–angiotensin–aldosterone system (RAAS) plays a crucial role in promoting cardiac fibrosis. Angiotensin II (Ang II) and aldosterone stimulate fibroblasts through dedicated receptors (including AT1), promoting fibroblast proliferation, production of reactive oxygen species and the synthesis of collagen and other ECM components [46].

Chronic activation of the Ang II-AT1 axis and mineralocorticoid signalling are correlated with an inflammatory response, upregulation of TGF-β, and maladaptive remodelling phenomena, justifying the clinical efficacy of RAAS-blocking drugs in reducing the progression of cardiac fibrosis [46,47].

Table 3 provides a concise overview of the major molecular signaling pathways involved in the development and progression of cardiac fibrosis.

### 5.5. Emerging Role of Transcriptional Regulators and Mitochondrial Metabolism

Recent evidence indicates that Serum Response Factor (SRF) represents a crucial transcriptional regulator in cardiac fibrosis, acting as an integrative node between mechanical, cytoskeletal, and profibrotic signals. SRF is strictly dependent on actin dynamics and cytoskeletal tension and controls the expression of genes involved in cell contractility, cytoskeletal organization and myofibroblast differentiation, including the expression of α-SMA and extracellular matrix components. Furthermore, SRF functionally interacts with key profibrotic pathways, with the TGF-β/SMAD pathway, amplifying its transcriptional response, and with YAP/TAZ-mediated mechano-transduction circuits, contributing to translate changes in extracellular matrix stiffness into adaptive or pathological fibroblastic responses. Experimental studies suggest that SRF overactivation in cardiac fibroblasts promotes collagen deposition and persistence of the fibrotic phenotype, indicating that SRF may represent a central regulatory node in myocardial fibrogenesis and a potential emerging therapeutic target [48]

In parallel, mitochondrial dysfunction is increasingly recognized as a key determinant of cardiac fibrotic remodeling. In activated fibroblasts, alterations in mitochondrial biogenesis, oxidative phosphorylation, and redox homeostasis favour a metabolic switch toward a highly synthetic and profibrotic phenotype. In this context, sirtuins, particularly SIRT1 and SIRT3, play a central role in maintaining metabolic homeostasis, regulating oxidative stress, and modulating the inflammatory response. Reduced sirtuin activity has been associated with increased myofibroblast activation and extracellular matrix deposition, while pharmacological modulation of these pathways appears to attenuate fibrotic remodelling in experimental models. Taken together, these data suggest that the integration of biomechanical signaling, transcriptional regulation, and cellular metabolism represents a key element in the progression of cardiac fibrosis [49,50].

**Table 3:** Major molecular signaling pathways involved in cardiac fibrosis.

Signalling Pathway	Activating Stimuli	Effects on Cardiac Fibroblasts	Histopathological Outcome
TGF-β/SMAD [33,34,35,36,37,38]	Inflammation, angiotensin II, tissue injury.	Fibroblast-to-myofibroblast transdifferentiation and increased extracellular matrix synthesis.	Diffuse collagen deposition and expansion of fibrotic tissue.
Wnt/β-catenin [39,40,41,42,43]	Chronic stress, ischaemic injury.	Fibroblast proliferation and acquisition of a synthetic phenotype.	Fibrotic expansion and altered myocardial architecture.
Hippo-YAP/TAZ [44,45]	Increased extracellular matrix stiffness and mechanical stress.	Mechanotransduction-driven fibroblast activation and persistence.	Progressive fibrosis and tissue stiffening.
RAAS [46,47]	Hypertension, heart failure, and neurohormonal activation.	Enhanced collagen synthesis, oxidative stress and profibrotic signalling.	Interstitial and perivascular fibrosis.
SRF [48]	Cytoskeletal tension, actin polymerization, ECM stiffness, TGF-β signaling.	Induces myofibroblast differentiation (↑ α-SMA), enhances collagen synthesis, and integrates mechano-transduction signals (interaction with TGF-β/SMAD and YAP/TAZ).	Increased interstitial and replacement fibrosis, persistent fibrotic phenotype.
Mitochondrial dysfunction/SIRT1–SIRT3 axis [49,50]	Oxidative stress, impaired mitochondrial biogenesis, metabolic imbalance, and aging-related signals.	Promotes profibrotic metabolic reprogramming, enhances myofibroblast activation and ECM deposition, reduced antifibrotic sirtuin signaling.	Progressive interstitial fibrosis, increased ECM accumulation and collagen cross-linking.

Data summarized from references [33,34,35,36,37,38,39,40,41,42,43,44,45,46,47,48,49,50].

## 6. Epigenetic Regulation and Non-Coding RNAs

In recent years, it has emerged that epigenetic regulation and non-coding RNAs play a central role in controlling fibroblast activation and the progression of cardiac fibrosis by modulating gene expression without altering the DNA sequence. These mechanisms contribute to the persistence of the myofibroblastic phenotype and the chronicity of fibrotic remodelling.

### 6.1. microRNA

MicroRNAs (miRNAs) are small noncoding RNAs that regulate gene expression at the post-transcriptional level and represent one of the main levels of control of cardiac fibrogenesis. Among these, miR-21 is one of the most studied miRNAs and is significantly overexpressed in activated cardiac fibroblasts. It promotes trans-differentiation into myofibroblasts and extracellular matrix deposition through the repression of antifibrotic genes and the indirect activation of the TGF-β/SMAD pathway. Experimental studies have demonstrated that miR-21 inhibition attenuates myocardial fibrosis and improves cardiac function in several preclinical models [51].

In contrast, miR-29 plays a predominantly antifibrotic role, negatively regulating the expression of genes encoding collagen (COL1A1, COL3A1), fibronectin, and other ECM proteins. The reduction in miR-29 observed in fibrotic myocardium is associated with an increase in extracellular matrix synthesis, while its experimental restoration limits fibrotic expansion [52,53].

Numerous other miRNAs contribute to the fine-tuning of cardiac fibrosis, including miR-133, miR-101, miR-125b, and miR-208, which modulate fibroblast proliferation, inflammation, and myofibroblast activation, underscoring the existence of a complex post-transcriptional control network [54,55].

### 6.2. lncRNAs

Long non-coding RNAs (lncRNAs) have emerged as important regulators of cardiac fibrosis, acting at multiple levels of gene regulation. Among these, lncRNA H19 has been associated with promoting fibroblast activation and increasing ECM deposition, partly through interaction with antifibrotic miRNAs and modulation of profibrotic pathways such as TGF-β and Wnt/β-catenin [56].

LncRNAs exert their effects through various mechanisms of action, including acting as microRNA sponges, modulating chromatin structure, and directly interacting with transcription factors or epigenetic complexes. This allows them to orchestrate stable transcriptional programs that favour the maintenance of the myofibroblastic phenotype in the fibrotic myocardium [57,58].

### 6.3. Classical Epigenetic Modifications

In addition to non-coding RNAs, classical epigenetic modifications play a fundamental role in the programming of cardiac fibroblasts. DNA methylation at the promoters of antifibrotic genes and histone acetylation/deacetylation directly influence chromatin accessibility and the transcription of genes involved in ECM production and the inflammatory response [59,60,61].

Alterations in the activity of epigenetic enzymes, such as DNA methyltransferases (DNMTs) and histone deacetylases (HDACs), have been associated with increased fibroblast activation and persistent pathological myocardial remodelling. Pharmacological interference with these mechanisms has shown antifibrotic effects in experimental models, suggesting new therapeutic perspectives [62,63,64].

## 7. Histopathological and Immunohistochemical Assessment of Cardiac Fibrosis: Potential and Limitations

Histopathological evaluation is a fundamental tool for the characterization of cardiac fibrosis, allowing the identification of collagen distribution patterns, extracellular matrix composition, and fibroblast activation status [15,16,17,18]. Traditional histological techniques, such as Masson’s trichrome staining and Picrosirius Red staining, allow a semi-quantitative and quantitative evaluation of collagen deposition and its structural organization. In particular, Picrosirius Red observed in polarized light allows the distinction between mature and immature collagen fibers, providing information on the dynamics of fibrotic remodelling [15,16,17,18].

Immunohistochemistry has further expanded the ability to characterize cardiac fibrosis, allowing the identification of activated fibroblasts and myofibroblasts through markers such as α-SMA, vimentin, and periostin [15,16,17]. α-SMA expression is commonly used to identify activated myofibroblasts, while periostin is considered a relatively more specific indicator of fibroblast activation associated with tissue remodelling [16,18]. Furthermore, mechano-transduction markers such as YAP/TAZ and molecules involved in extracellular matrix deposition and maturation, including LOX, can provide functional information on the biological state of fibrosis [15,19].

However, each marker has significant interpretive limitations. α-SMA expression is not exclusive to myofibroblasts and can also be observed in vascular smooth muscle cells and some pericyte populations, reducing its diagnostic specificity [15,16]. Similarly, quantification of collagen by histological staining does not allow distinction between active fibrosis and established or cicatricial fibrosis. Furthermore, the collagen density observed microscopically does not necessarily reflect the biomechanical stiffness of the tissue, which also depends on the degree of cross-linking and the three-dimensional organization of the extracellular matrix [17,18,19,20].

Further interpretive challenges arise from the dynamic nature of fibrotic remodelling. Histological analysis represents a static assessment of a highly dynamic biological process influenced by the clinical context, the etiology of the heart disease, and the temporal phase of the disease [15,17]. Furthermore, variability in tissue sampling and regional heterogeneity of fibrosis may limit diagnostic accuracy, especially in biopsy specimens. Integrating histopathological analysis with digital quantitative techniques and molecular and omics data therefore represents a promising approach to improve the phenotypic characterization of cardiac fibrosis and its correlation with clinical outcomes [15,16,17,18,19,20].

Overall, although histopathological evaluation remains the gold standard for the morphological characterization of cardiac fibrosis, its interpretation requires an integrated approach that simultaneously considers structural, molecular, and clinical data [15,16,17,18,19,20,21,22].

## 8. Integrating Histopathological Assessment and Molecular Data in Cardiac Fibrosis

In addition to morphological characterization, histopathological evaluation plays a crucial role in the functional interpretation of molecular and omics data in cardiac fibrosis [15,16,17,18]. High-resolution technologies, including transcriptomic, proteomic, and epigenomic approaches, have significantly expanded our understanding of the pathogenic mechanisms of fibrosis [15,16,17,18,19,20]. However, such methodologies often reflect cellular activation states that do not necessarily translate into permanent structural changes in myocardial tissue. In this context, histological analysis allows us to distinguish between transient molecular activation and established fibrotic remodelling, providing an essential level of interpretation to contextualize molecular findings [15,17].

Specifically, the presence of overexpression of profibrotic genes or mechano-transduction pathways identified using omics approaches can be supported by histological demonstration of extracellular matrix deposition and myofibroblast activation [15,16,17,18,19,20]. Conversely, the absence of significant morphological correlates in the presence of molecular alterations suggests that such signals may represent early or potentially reversible phases of tissue remodelling [17,18,19,20]. Furthermore, histopathological evaluation allows us to analyse the spatial distribution of fibrosis, distinguishing between interstitial, replacement, or perivascular patterns, aspects that cannot be completely reconstructed from molecular data obtained from homogenized tissues [15,18].

A further contribution of the anatomopathological analysis concerns the possibility of identifying cellular and microenvironmental heterogeneity within the fibrotic tissue, allowing us to correlate molecular expression with specific cell populations and with the local structural context [16,17,18,19,20]. This integration is particularly relevant because omics studies may overestimate the contribution of specific molecular pathways without considering the complex interplay between inflammatory cells, fibroblasts, vascular cells, and cardiomyocytes [15,16,17,18].

Despite these strengths, the pathology has intrinsic limitations in assessing the dynamic processes of fibrosis. Histological analysis represents a static photograph of a highly dynamic and temporal biological phenomenon [17,18,19,20]. Therefore, the integration of histopathological, molecular, and clinical data represents an indispensable approach for a comprehensive understanding of fibrotic remodelling and to improve the translation of biological knowledge into diagnostic and prognostic applications [15,16,17,18,19,20,21,22].

## 9. Correlation Between Molecular Evidence, Histopathological Diagnosis and Sudden Cardiac Death

The molecular alterations that characterise cardiac fibrosis have a clear counterpart at the tissue level, rendering histopathological diagnosis a key element in linking pathogenic mechanisms to clinical manifestations, including sudden cardiac death (SCD). Persistent fibroblast activation and their transdifferentiation into myofibroblasts are reflected histologically by increased extracellular matrix deposition, disruption of myocardial architecture, and enhanced tissue stiffness. These changes promote electrical instability and the formation of an arrhythmogenic substrate, thereby increasing the risk of SCD [65,66,67,68,69].

Several immunohistochemical markers are particularly valuable for the characterisation of fibrosis and pathological cardiac remodelling. Expression of α-smooth muscle actin (α-SMA) identifies activated myofibroblasts, while assessment of type I and type III collagen and fibronectin enables definition of the composition and organisation of the extracellular matrix. More recently, immunohistochemical evaluation of YAP/TAZ has facilitated the correlation between cellular mechano-transduction, matrix stiffness, fibroblast expansion, and progression of fibrosis [11,29].

The integration of digital morphometry into routine histopathological practice has further enhanced the objective quantification of fibrosis. Image analysis software allows precise measurement of collagen deposition, thereby reducing interobserver variability and overcoming the limitations of semi-quantitative visual assessment. In this setting, the application of artificial intelligence and machine-learning algorithms is emerging as a promising approach for automated histological analysis, enabling more accurate risk stratification and improved correlations between structural alterations and clinical outcomes, including susceptibility to fatal arrhythmic events [21].

Within this complex framework, the cardiovascular pathologist plays a central role as a bridge between molecular research and clinical practice, integrating histological, immunohistochemical, and quantitative data with an in-depth understanding of the molecular pathways underlying fibrosis. This multidisciplinary approach is essential for the correct interpretation of the structural substrates of lethal arrhythmias and for the development of increasingly refined diagnostic and prognostic criteria in the assessment of sudden cardiac death risk [70].

Table 4 provides an overview of the main diagnostic and prognostic implications associated with different patterns of cardiac fibrosis.

### Cardiac Fibrosis, Arrhythmogenesis, and Clinical Risk Stratification

Cardiac fibrosis is increasingly recognized as a potentially arrhythmogenic structural substrate, capable of altering the propagation of the myocardial electrical impulse and promoting the onset of malignant ventricular arrhythmias. However, despite biological plausibility and growing translational interest, evidence directly linking fibrotic patterns to SCD risk remains incomplete and sometimes heterogeneous.

ECM deposition, regulated by pathways such as TGF-β/SMAD, Wnt/β-catenin and Hippo-YAP/TAZ [15,16,17,18,19,20], determines discontinuity of the myocardial tissue, slowing of electrical conduction and increased electrophysiological heterogeneity, creating substrates favourable to the formation of re-entry circuits.

Experimental studies on mechano-transduction and fibroblast activation [17,18,19,20] suggest that the spatial distribution of fibrosis, rather than its overall extent, represents a critical determinant of arrhythmic risk. Diffuse and patchy interstitial fibrosis can, in fact, promote dispersion of conduction and inhomogeneity of myocardial electrical recovery, increasing the electrical vulnerability of the ventricle.

Pathologically, the identification of fibrotic patterns provides relevant information on potential electrical instability. Replacement fibrosis, frequently observed in post-ischemic or post-inflammatory remodelling [15,18], is associated with the formation of re-entry loops, whereas diffuse interstitial fibrosis may contribute to more subtle but extensive conduction disturbances. However, the sole quantification of fibrotic extension does not appear sufficient to predict arrhythmic risk, since factors such as ECM composition, the degree of collagen cross-linking and the interaction with cellular and ionic alterations significantly modulate the electrical stability of the myocardium [15,21].

In recent years, the integration of pathological data and advanced cardiac imaging has strengthened our understanding of the role of fibrosis in risk stratification. In particular, the correlation between fibrotic patterns and structural and functional alterations, already discussed in the previous sections in relation to mechano-dependent remodelling [17,18,19,20], suggests that the combination of morphological and functional data may improve the selection of patients eligible for preventive strategies. However, the relationship between the amount of fibrosis and arrhythmic risk varies significantly in different clinical settings, and there are currently no universally validated quantitative thresholds.

Available evidence indicates that cardiac fibrosis represents a modulator of arrhythmic risk rather than an isolated determinant of SCD. Therefore, the pathological assessment of fibrosis should be interpreted within a multimodal approach that integrates structural, molecular and clinical data, in order to improve prognostic stratification.

## 10. Therapeutic Implications

Cardiac fibrosis represents an important therapeutic target in the management of cardiovascular disease, as excessive deposition of extracellular matrix is associated with mechanical dysfunction, arrhythmogenesis, and progression to heart failure. Despite the absence of approved therapies that specifically and exclusively target cardiac fibrosis, a range of emerging antifibrotic strategies has been developed in recent years across multiple molecular and pharmacological domains.

The TGF-β pathway is a central driver of fibroblast activation and collagen synthesis. Inhibition of TGF-β signalling represents a promising strategy for limiting cardiac fibrosis in several experimental models. Pharmacological agents and biological approaches targeting this pathway, including small molecules and antiTGF-β antibodies, are currently under preclinical investigation, as they interfere with profibrotic signal transduction and reduce the expression of key extracellular matrix genes in activated fibroblasts [71].

Modulation of the RAAS remains fundamental in the treatment of heart failure and in the regulation of cardiac remodelling. Angiotensin-converting enzyme (ACE) inhibitors, angiotensin receptor blockers (ARBs), and mineralocorticoid receptor antagonists (MRAs) attenuate the profibrotic stimulation induced by angiotensin II and aldosterone, thereby reducing collagen deposition and improving ventricular function. In addition, newer therapeutic classes, such as angiotensin receptor-neprilysin inhibitors (ARNIs) and sodium-glucose co-transporter 2 (SGLT2) inhibitors, have demonstrated indirect antifibrotic effects in patients with heart failure, with evidence of reduced interstitial collagen accumulation and modulation of fibrotic markers in human cardiac tissue [72].

Epigenetic regulation and n-c RNAs represent innovative therapeutic frontiers in the treatment of cardiac fibrosis. Alterations in DNA methylation, histone acetylation and deacetylation, and chromatin remodelling critically influence fibroblast activation and profibrotic gene expression. In preclinical studies, inhibitors of histone deacetylases (HDACs), DNA methyltransferases (DNMTs), histone methyltransferases (HMTs), and other epigenetic regulators have demonstrated the ability to attenuate fibroblast activation and extracellular matrix deposition, offering potentially reversible and targeted antifibrotic strategies [73].

In parallel, RNA-based therapeutic approaches, including antisense oligonucleotides and microRNA (miRNA) mimics or inhibitors, aim to correct post-transcriptional dysregulation of RNAs involved in fibrogenesis. For instance, suppression of profibrotic miRNAs or enhancement of antifibrotic miRNAs has been shown to reduce fibroblast-to-myofibroblast transdifferentiation and collagen production in experimental models [74].

Despite these advances, significant limitations continue to hinder the clinical translation of antifibrotic therapies. The complexity of profibrotic signalling networks, interspecies variability between animal models and humans, off-target effects, and challenges in the delivery of RNA-based therapies remain major obstacles. Moreover, to date, no therapy has been specifically approved for the treatment of isolated cardiac fibrosis, and most antifibrotic effects are achieved indirectly through treatments for broader cardiovascular conditions [71].

Future perspectives include the development of agents targeting critical molecular pathways, the integration of combination strategies encompassing pharmacological, genetic, and cellular approaches, the identification and validation of prognostic biomarkers, and the implementation of precision medicine frameworks to stratify patients according to their molecular fibrosis profiles, with the aim of maximising therapeutic efficacy while minimising adverse effects.

## 11. Unresolved Issues in Cardiac Fibrosis: Biological and Diagnostic Implications

Despite significant advances in understanding the molecular mechanisms of cardiac fibrosis, several fundamental questions remain unresolved and represent important limitations in the clinical translation of current knowledge.

The repeated failure of antifibrotic therapies in clinical trials suggests that cardiac fibrosis is an extremely complex and multifactorial biological process. Therapeutic approaches targeting single molecular pathways, such as the TGF-β/SMAD pathway [15], Wnt/β-catenin signaling [16], the Hippo-YAP/TAZ axis [17,18,19,20] and the RAAS [21,22], have shown partial or non-durable benefits, likely due to the redundancy of profibrotic circuits, the interaction between convergent pathways and the cellular heterogeneity of the myocardial microenvironment. Furthermore, fibrosis may initially represent an adaptive response aimed at maintaining the structural integrity of the myocardium and its indiscriminate inhibition could therefore interfere with physiological repair mechanisms [15,18].

A further controversy concerns the pathogenetic role of cardiac fibrosis, which can constitute, depending on the clinical context, either a primary driver of myocardial dysfunction or an epiphenomenon secondary to processes such as chronic inflammation, ischemia or hemodynamic overload. Fibroblast activation and ECM deposition may precede and directly contribute to ventricular stiffness and diastolic dysfunction; however, in other settings, collagen accumulation appears predominantly as a reparative response to pre-existing myocyte damage [15,16,17,18,19,20]. The distinction has diagnostic and therapeutic relevance, but it is complex when using exclusively molecular or exclusively morphological approaches.

A further critical aspect concerns the temporal reversibility of cardiac fibrosis. Experimental data suggest that early forms of interstitial fibrosis, characterized by quantitative alterations of the ECM with limited collagen cross-linking, may be at least partially reversible [17,18,19,20]. As remodelling progresses, however, increased collagen maturation and cross-linking, persistent myofibroblast activation, and matrix stabilization make fibrosis progressively less susceptible to regression [15,21]. In this situation, pathological assessment has intrinsic limitations as it provides a static snapshot of a dynamic biological process and does not allow for a definitive distinction between actively evolving fibrosis and established cicatricial fibrosis, nor for a precise definition of the therapeutic timeframe.

Overall, these issues, already implicitly emerged in the discussion of molecular pathways and histopathological patterns [15,16,17,18,19,20,21,22], underline the need for a truly integrated approach combining molecular data, quantitative histopathological analysis, mechano-biological assessments and longitudinal clinical correlations.

**Figure 3:**
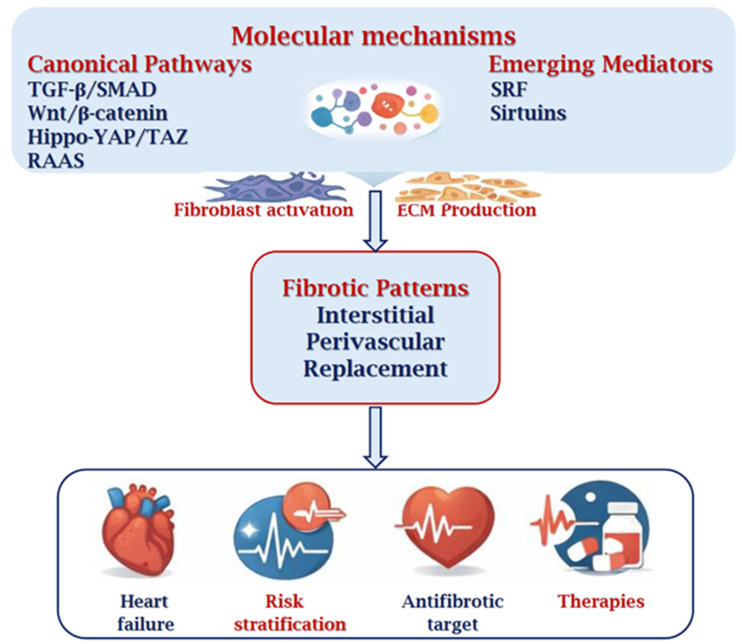
Integrated schematic representation of molecular pathways, histopathological patterns, and clinical consequences of cardiac fibrosis. The diagram illustrates the major profibrotic signaling pathways involved in myocardial fibrogenesis, including TGF-β/SMAD, RAAS (angiotensin II and aldosterone), Wnt/β-catenin, and Hippo-YAP/TAZ signaling, as well as emerging regulators such as SRF and mitochondrial dysfunction/SIRT pathways. These molecular mechanisms converge on cardiac fibroblast activation and transdifferentiation into α-SMA-positive myofibroblasts, leading to excessive extracellular matrix deposition and collagen cross-linking. The resulting structural remodeling generates distinct histopathological patterns (interstitial, perivascular, and replacement fibrosis), which contribute to myocardial stiffening, electrical conduction abnormalities, heart failure, ventricular arrhythmias, and SCD.

Figure 3 summarizes the major molecular pathways involved in myocardial fibrogenesis, including TGF-β/SMAD, RAAS, Wnt/β-catenin, Hippo-YAP/TAZ, and emerging regulators such as SRF, mitochondrial dysfunction, and sirtuins. The resulting fibroblastic activation determines ECM deposition and the formation of different fibrotic patterns identifiable by histopathological and immunohistochemical analysis. Structural alterations of the matrix contribute to mechanical and electrophysiological dysfunctions, with clinical consequences such as heart failure, ventricular arrhythmias, and SCD.

## 12. Conclusions

Cardiac fibrosis represents a complex and dynamic process integrating mechanical, inflammatory, neurohormonal, and epigenetic stimuli, culminating in profound alterations of myocardial architecture and cardiac function. The different morphological patterns of fibrosis, interstitial, perivascular, and replacement, are not merely histological observations but reflect distinct underlying cellular and molecular mechanisms with significant pathophysiological and clinical implications.

From a pathologist’s perspective, the activation of fibroblasts and their transdifferentiation into myofibroblasts constitute the pivotal event in cardiac fibrogenesis. This process is orchestrated by highly integrated signalling pathways, including TGF-β/SMAD, Wnt/β-catenin, and Hippo–YAP/TAZ, as well as by the actions of neurohormonal mediators such as angiotensin II and aldosterone. In addition, epigenetic and post-transcriptional regulation, mediated by microRNAs, lncRNAs, and chromatin modifications, contributes to the stabilization of the profibrotic phenotype and the chronicity of pathological remodelling.

The histopathological manifestation of these mechanisms, including collagen deposition, an increased collagen I/III ratio, the presence of α-SMA-positive myofibroblasts, and enhanced cross-linking of the extracellular matrix, represents the point of convergence between molecular biology and morphological diagnosis. In this context, the integration of advanced immunohistochemistry, digital morphometric analysis, and computational approaches based on artificial intelligence is progressively reinforcing the role of cardiovascular pathology as a central discipline in risk stratification, including the assessment of arrhythmogenic substrates and the risk of sudden cardiac death.

Finally, a thorough understanding of the molecular mechanisms underlying cardiac fibrosis provides the rationale for developing more targeted antifibrotic therapeutic strategies. Although clinical translation remains challenging, the identification of critical molecular nodes and histo-molecular biomarkers offers tangible prospects for a precision medicine approach, in which the pathologist plays a central role in linking basic research with clinical practice. In conclusion, only through an integrated approach that combines morphology, molecular biology, and clinical practice can the impact of cardiac fibrosis on cardiovascular diseases and their most serious outcomes be effectively addressed.

## Figures and Tables

**Table 1:** Histopathological patterns of cardiac fibrosis and underlying molecular mechanisms.

Type of Fibrosis	Histological Distribution	Cellular Mechanisms	Molecular Pathways	Functional Implications
Interstitial [7,8,9,10]	Diffuse between cardiomyocytes, endomysial and perimysial expansion.	Persistent activation of resident fibroblasts.	TGF-β/SMAD, miR-29 downregulation, YAP/TAZ.	Increased myocardial stiffness, diastolic dysfunction, HFpEF.
Perivascular [11,12]	Accumulation of ECM around intramyocardial vessels.	Activation of perivascular fibroblasts and chronic inflammatory response.	RAAS, Wnt/β-catenin, TGF-β.	Microvascular dysfunction, subclinical ischemia.
Replacement [13,14]	Post-necrotic scar areas.	Repair response with irreversible loss of cardiomyocytes.	TGF-β, inflammation, and collagen cross-linking.	Reduced contractility, arrhythmogenic substrate.

Data summarized from references [7,8,9,10,11,12,13,14].

**Table 2:** Histological and immunohistochemical markers of cardiac fibrosis.

Marker	Technique	Biological Significance	Diagnostic Relevance
Type I collagen [15,18,19]	Immunohistochemistry/Picrosirius Red	Rigid extracellular matrix component associated with increased myocardial stiffness.	Indicator of advanced and irreversible fibrosis.
Type III collagen [15,18,19]	Immunohistochemistry/Picrosirius Red	More elastic extracellular matrix component.	Marker of early or potentially reversible fibrosis.
α-smooth muscle actin (α-SMA) [16]	Immunohistochemistry	Myofibroblast activation and contractile phenotype.	Marker of active fibrotic remodelling.
Fibronectin [20,21]	Immunohistochemistry	Extracellular matrix remodelling and fibroblast activation.	Indicator of ongoing fibrotic progression.
Lysyl oxidase (LOX) [17,18]	Immunohistochemistry	Collagen cross-linking and matrix stabilization.	Contributor to increased tissue stiffness and resistance to degradation.
YAP/TAZ [17,20]	Immunohistochemistry (nuclear localization)	Mechanotransduction and response to extracellular matrix stiffness.	Marker of mechanically driven fibroblast activation.

Data summarized from references [15,16,17,18,19,20,21,22].

**Table 4:** Diagnostic and prognostic implications of cardiac fibrosis.

Fibrotic Feature	Histopathological Evidence	Clinical and Prognostic Implications
Active fibrosis (α-SMA–positive myofibroblasts). [65,66,67]	Persistence of activated myofibroblasts within the interstitium.	Disease progression and potentially reversible remodelling.
Disorganized collagen architecture. [65,68,69]	Irregular and non-aligned collagen fibers.	Electrical conduction heterogeneity and increased arrhythmogenic risk.
Extensive replacement fibrosis. [65,66,67,68,69]	Dense collagenous scars replacing myocardial tissue.	Substrate for ventricular arrhythmias and SCD.
Perivascular fibrosis. [29]	Extracellular matrix accumulation surrounding intramyocardial vessels.	Chronic myocardial ischemia and microvascular dysfunction.
Increased extracellular matrix stiffness. [11,21,70]	Enhanced collagen cross-linking.	Diastolic dysfunction, HFpEF, and arrhythmias.

Data summarized from references [11,21,29,65,66,67,68,69,70].

## Data Availability

No new data were created or analyzed in this study. Data sharing is not applicable to this article.

## Abbreviations

ACE	Angiotensin-converting enzyme
α-SMA	Alpha-smooth muscle actin
ARB	Angiotensin receptor blocker
ARNI	Angiotensin receptor–neprilysin inhibitor
AT1	Angiotensin II type 1 receptor
CKD	Chronic kidney disease
DNMT	DNA methyltransferase
ECM	Extracellular matrix
HDAC	Histone deacetylase
HFpEF	Heart failure with preserved ejection fraction
HMT	Histone methyltransferase
IHC	Immunohistochemistry
IL	Interleukin
lncRNA	Long non-coding RNA
LOX	Lysyl oxidase
MAPK	Mitogen-activated protein kinase
miRNA	MicroRNA
MRA	Mineralocorticoid receptor antagonist
n-c RNAs	Non-coding RNAs
PDGF	Platelet-derived growth factor
PDGFR-α	Platelet-derived growth factor receptor alpha
RAAS	Renin–angiotensin–aldosterone system
ROCK	Rho-associated protein kinase
SCD	Sudden cardiac death
SGLT2	Sodium–glucose co-transporter 2
SIRT	Sirtuin
SMAD	Mothers against decapentaplegic homolog proteins
SRF	Serum response factor
TAZ	Transcriptional coactivator with PDZ-binding motif
TGF-β	Transforming growth factor beta
TNF-α	Tumor necrosis factor alpha
Wnt	Wingless/Integrated signaling pathway
YAP	Yes-associated protein
